# Adenovirus Vectors Expressing Eight Multiplex Guide RNAs of CRISPR/Cas9 Efficiently Disrupted Diverse Hepatitis B Virus Gene Derived from Heterogeneous Patient

**DOI:** 10.3390/ijms221910570

**Published:** 2021-09-29

**Authors:** Yuya Kato, Hirotaka Tabata, Kumiko Sato, Mariko Nakamura, Izumu Saito, Tomoko Nakanishi

**Affiliations:** 1 Laboratory of Virology, Institute of Microbial Chemistry (BIKAKEN), Microbial Chemistry Foundation, Shinagawa-ku, Tokyo 141-0021, Japan; Yuya_Kato@kirin.co.jp (Y.K.); hirotaka.tabata0716@gmail.com (H.T.); m.nakamura.ou@juntendo.ac.jp (M.N.); nakanishi-t@juntendo.ac.jp (T.N.); 2 Laboratory of Molecular Genetics, Institute of Medical Science, University of Tokyo, Minato-ku, Tokyo 108-8639, Japan; lua.do.gelo@gmail.com; 3 Center for Biomedical Research Resources, Juntendo University Graduate School of Medicine, Tokyo 113-8421, Japan; 4 Department of Physiology, Juntendo University Graduate School of Medicine, Tokyo 113-8421, Japan

**Keywords:** CRISPR/Cas9, genome editing, adenovirus vector, multiplex guide RNAs, hepatitis B virus (HBV), hepatocellular carcinoma, gene therapy

## Abstract

Hepatitis B virus (HBV) chronically infects more than 240 million people worldwide, causing chronic hepatitis, cirrhosis, and hepatocellular carcinoma (HCC). Genome editing using CRISPR/Cas9 could provide new therapies because it can directly disrupt HBV genomes. However, because HBV genome sequences are highly diverse, the identical target sequence of guide RNA (gRNA), 20 nucleotides in length, is not necessarily present intact in the target HBV DNA in heterogeneous patients. Consequently, possible genome-editing drugs would be effective only for limited numbers of patients. Here, we show that an adenovirus vector (AdV) bearing eight multiplex gRNA expression units could be constructed in one step and amplified to a level sufficient for in vivo study with lack of deletion. Using this AdV, HBV X gene integrated in HepG2 cell chromosome derived from a heterogeneous patient was cleaved at multiple sites and disrupted. Indeed, four targets out of eight could not be cleaved due to sequence mismatches, but the remaining four targets were cleaved, producing irreversible deletions. Accordingly, the diverse X gene was disrupted at more than 90% efficiency. AdV containing eight multiplex gRNA units not only offers multiple knockouts of genes, but could also solve the problems of heterogeneous targets and escape mutants in genome-editing therapy.

## 1. Introduction

Hepatitis B virus (HBV) causes chronic hepatitis, liver cirrhosis, and hepatocellular carcinoma (HCC). Approximately 250 million people worldwide are persistently infected with this virus [1] and more than 850,000 die each year (World Health Organization (2019) factsheet, https://www.who.int/en/news-room/fact-sheets/detail/hepatitis-b, accessed on 28 September 2021). Current anti-HBV treatments with either nucleoside/nucleotide analogs or interferon-α cannot eliminate the virus, and relapse is common [2]. Consequently, there is an urgent need to cure chronic HBV patients in a fundamentally different way.

Chronic HBV infection is a major risk factor for the development of HCC, accounting for 60% of cases worldwide [3]. Integrated viral DNA has been reported in approximately 85% of HBV-related HCC cases [4,5,6]. The HBV X gene is often integrated into a chromosome in HCC cases [7] and correlated with HCC development and progression [8,9,10,11].

Genome editing approaches that directly target the HBV genome have been proposed as potential curative therapies [12,13]. Cas9 derived from *Streptococcus pyogenes* (spCas9) recognizes both PAM sequences of NGG and the upstream target sequence of 20 nucleotides (nt) specified by a single guide RNA (sgRNA; gRNA hereafter), and it cleaves the target DNA. The same therapeutic vector is probably effective for both chronic hepatitis and HCC because the vector requires only the nucleotide sequences of the target, present in both the free virus genome and the viral DNA integrated into a chromosome. However, a key problem for gene-editing therapy against HBV-associated diseases is that the HBV genome is extremely diverse [14,15]. Even in the same genotype, the sequences of HBV genomes diverge by up to 8% (i.e., 8 nt changes per 100 bp) and differ from patient to patient. Moreover, in chronic hepatitis, single patients possess diverse HBV genomes called quasispecies [16,17,18]. Drug-resistant mutant HBVs emerge within a short time because a few resistant genome clones are already present and quickly become a major population by selective growth [17].

In genome-editing applications, 20 bp recognition sequences must be identical to that of gRNA used for therapy. If two gRNAs are available, they produce an irreversible deletion and the target gene is effectively disrupted. However, in this strategy, 40 nt sequences must be identical. Moreover, inefficient cleavage of one of the gRNA targets would result in viral escape [19,20], suggesting that two gRNAs are probably insufficient to inhibit escape. De Silva Feelixge et al. [21] suggested that multiplex target sites that encompass a highly conserved viral protein-coding region may prevent resistance. However, currently, no vector systems are available to resolve this problem.

Replication-deficient or E1-deleted adenovirus vector (AdV) is one of the most efficient gene delivery systems, having a broad range of cell and tissue types [22]. This vector can transduce DNA to both dividing and nondividing cells and confers high expression. AdV does not integrate into a cellular chromosome and disappear after genome editing, which is an advantage over lentiviral vectors. Its capacity is 8 kb, sufficient to contain full spCas9 expression units, and it enables a double-nicking strategy to be used (see Section 3), in contrast to AAV vectors. These features are advantageous for CRISPR/Cas9 approaches [23]. Although high immunogenicity is considered a major drawback of this vector, this problem may be resolved using an AdV system, which showed low immunogenicity and long-term expression for six months in immunocompetent mice [24,25]. However, preexisting adaptive immunity to Cas9 protein is present in human population at a high frequency, which could reduce the efficacy of gene therapy using Cas9 [26,27,28].

We recently developed AdVs that contain four multiplex gRNA units consisting of two double-nicking units, and showed that they can be amplified without a lack of the gRNA expression units for in vivo experiments [29]. Here, we show that eight multiplex gRNA units inserted in the AdV genome were completely stable. As a model of HCC, we used a cell line containing the HBV X gene in a chromosome. We attempted to disrupt the X gene using an AdV expressing eight multiplex gRNAs. The HBV X gene was not cleaved at the sites of four targets out of eight due to sequence mismatches but was cleaved at the remaining four target sites, causing irreversible deletions. Consequently, the X gene was efficiently disrupted. These results suggested that this AdV could disrupt HBV X or other genes derived from many other heterogeneous patients with HCC and chronic hepatitis infected with HBV of different genotypes.

## 2. Results

### 2.1. Production of AdVs Expressing Multiplex gRNAs

**Figure 1:**
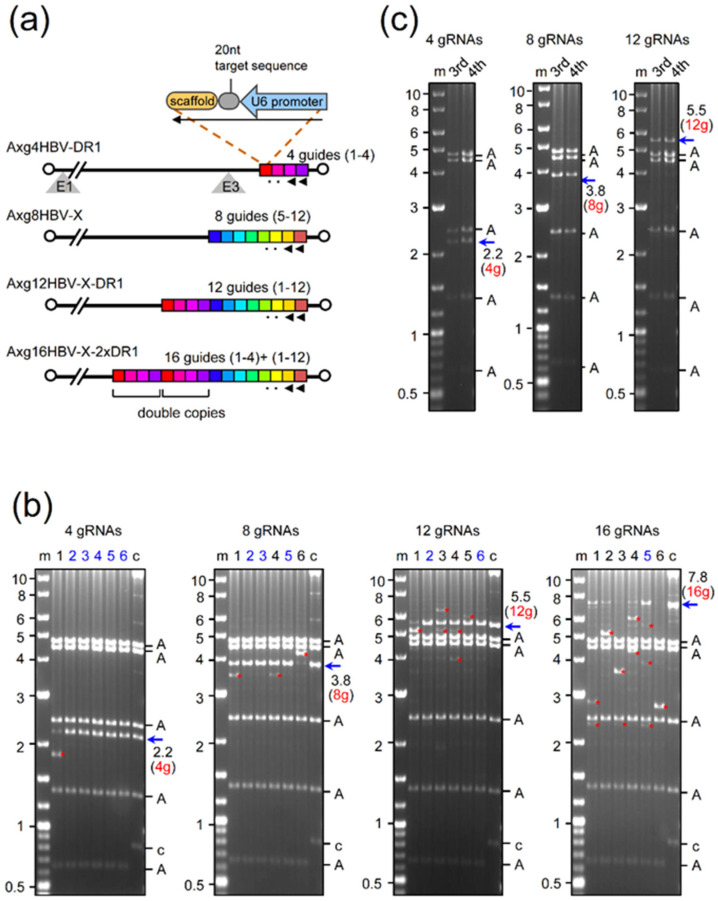
Production of highly multiplex AdVs. (**a**) Structures of AdVs. Each colored box corresponds to one gRNA expression unit. (**b**) The BspEI-cleaved AdV genome of the second viral stock. Lane m, marker; lanes 1–6, clone number; lane c, the parent cosmid containing the AdV genome. A, AdV fragment derived from the parent cosmid identical to the vector backbone; c, the fragment of the cosmid–AdV junction in the cosmid. A blue arrow shows the AdV DNA fragment containing multiplex gRNA units. Asterisk, a band of the DNA fragment containing fewer or more gRNA units. The total cellular DNA of 293 cells infected with AdV of the first stock was digested with BspEI and analyzed by agarose gel electrophoresis. The picture shown as 4 gRNAs originates from Figure 2b of reference [29]. (**c**) The BspEI-cleaved AdV genome derived from infected cells with the third and fourth stocks.

The structures of AdVs that contain multiplex gRNA expression units are shown in Figure 1. The unit, approximately 400 bp in length, consists of the U6 promoter of approximately 270 bp, the target sequences of 20 nt, and the scaffold sequence of approximately 90 nt (upper right), together with the connecting region of approximately 20 bp complementary to the sequencing primers. We have reported the AdV Axg4HBV-DR1 (Figure 1a, the first AdV), containing four gRNA expression units in tandem, that targets the C-terminal region of the HBV X gene near DR1 (the right end of the AdV genome shown as red to purple units in the figure). To examine whether more gRNA units inserted in the AdV genome can be stably maintained, we attempted to construct AdVs bearing eight, twelve, and sixteen gRNA units in tandem (Figure 1a, second to fourth AdVs). The second AdV, Axg8HBV-X, contains eight gRNA units, targeting the central coding region of the HBV X gene (blue to orange units in the second AdV genome). The third AdV, Axg12HBV-X-DR1, conta ins all twelve gRNA units, targeting both regions (red to orange units). By chance, we obtained a cosmid containing the AdV genome of Ax16HBV-X-2xDR1 that bears 16 gRNA units, which consists of two sets of four units targeting DR1 (double copies of red to purple array), together with eight units targeting the X region (blue to orange array).

We have reported that AdV Axg4HBV-DR1 was obtained with no detectable lack of the four gRNA units present in the AdV genome (Figure 1b, the first, leftmost panel is shown as “4 gRNAs”) [29]. The 293 cells were transfected with the DNA of the AdV genome containing four gRNA units, and the total cellular DNAs of 293 cells infected with the first viral stock of this AdV were prepared and digested with BspEI to examine the copy numbers of the gRNA units. Five AdV clones out of six produced a 2.2 kb fragment, which contained four intact gRNA units, and no band derived from deleted units was detected (lanes 2–6, blue arrow of “2.2 (4g)”). In contrast, clone 1 produced a 1.8 kb band instead of a 2.2 kb band (lane 1). The size exactly corresponds to that containing three gRNA units, showing that one unit was deleted through homologous recombination (the extra bands other than those containing the expected intact copies of gRNA units are marked by red asterisks, hereafter) [29]. The third and fourth stocks were prepared, and BspEI assay was performed (Figure 1c, panel of “4 gRNAs,”). The intensity of the 2.2 kb band containing four gRNA units was similar to that of the higher band shown as “A” derived from the vector backbone, suggesting that the copy numbers of the two DNA fragments are comparable. In our experience, the four gRNA units were always stably maintained with no apparent lack in the fourth viral stock, which is sufficient for in vivo experiments. The expression of all gRNAs using the AdV containing four gRNAs was directly confirmed by an AdV containing the same structure in our previous report [29].

To examine whether eight or more gRNA units can be stably maintained in the AdV genome, three other AdV DNAs of Axg8HBV-X, Axg12HBV-X-DR1, and Axg16HBV-X-2xDR1 were assayed, the results of which are shown in Figure 1b as panels of “8 gRNAs”, “12 gRNAs”, and “16 gRNAs”, respectively. We found that the eight gRNA units in the AdV genome were maintained apparently intact in three clones out of six (“8 gRNAs,” lanes 2, 3, and 5, 3.8 kb band shown by a blue arrow) because no deleted bands were detected in these lanes. Clone 1 and clone 4 (lanes 1 and 4) produced the main 3.8 kb bands as well as lower extra bands of 3.4 kb (two red asterisks), suggesting that these clones also contain the AdV missing one of the eight units. Interestingly, clone 6 (lane 6) mostly produced a higher extra band of 4.2 kb corresponding to nine gRNA units, suggesting that a homologous recombination increasing one unit occurred at an early stage of AdV genome replication.

Two AdV clones out of six contained a considerable amount of the intact array of twelve gRNA units (“12 gRNAs,” lanes 2 and 6, the 5.5 kb band shown by red 12 g and a blue arrow). The copy numbers of the intact twelve units were more than half of those derived from the vector backbone (two As below the blue arrow). Most of the AdVs contained fewer or even more copies of the twelve units (bands shown by red asterisks). For AdVs containing sixteen gRNA units (panel “16 gRNAs”), the lanes containing a 7.8 kb band bearing sixteen units were mostly lost, but one clone still maintained a significant amount of AdVs bearing sixteen intact gRNA units (lane 5).

Next, we examined whether AdVs containing eight and twelve gRNA units can be amplified with no lack of the multiplex gRNA units or not using clone 2 (Figure 1b, “8 gRNAs,” lane 2) and clone 2 (“12 RNAs,” lane 2), respectively. As for eight-unit AdV, the eight gRNA units appeared to be maintained with no significant lack in the third and fourth stocks, considering the intensity of the bands among the AdV bands (compare Figure 1b, “8gRNAs,” lane 2 with Figure 1c, “8gRNAs,” lanes 3 and 4). The results suggest that, once isolated, the eight gRNA units were stably amplified. To date, we have constructed six AdVs that contain eight gRNA units, and in all cases the eight units were stably maintained even in the fifth stock and in purified stocks sufficient for in vivo experiments, confirming high stability during amplification. As for twelve-unit AdV, the ratios of twelve gRNA units in the third and four stocks were significantly lower than that in the second stock (compare Figure 1b, “12gRNAs” lane 2 with Figure 1c, “12gRNAs”).

### 2.2. Disruption of HBV-X Gene Derived from a Heterogeneous Patient Using AdVs Expressing Eight and Twelve gRNAs

**Figure 2:**
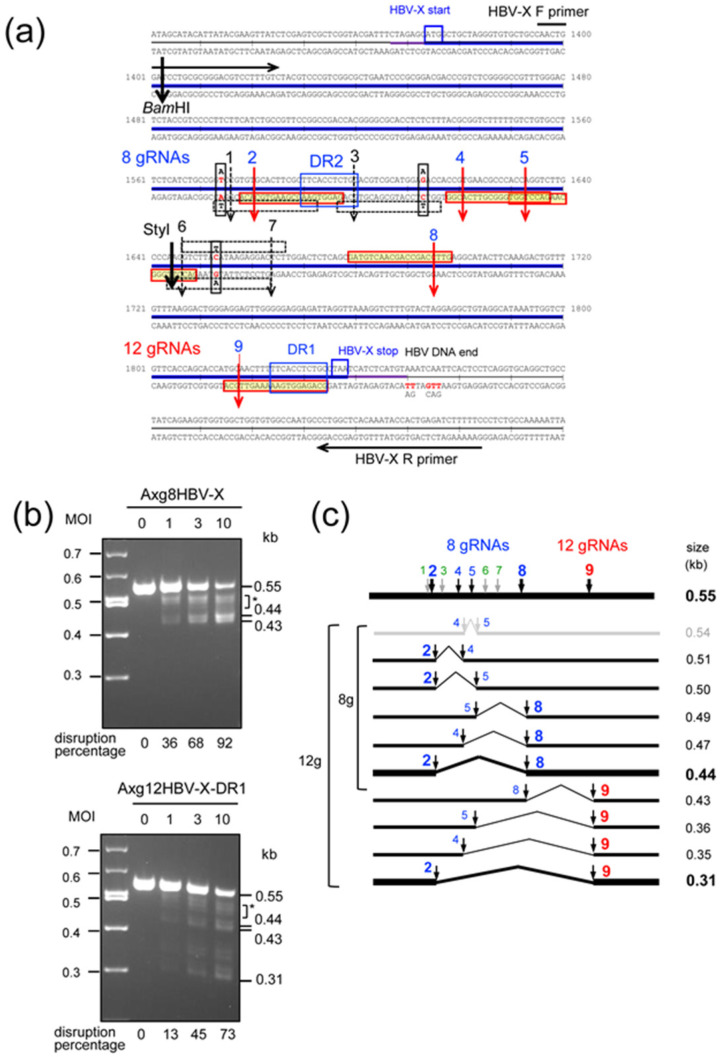
Disruption of the HBV X gene derived from a different patient using AdV expressing 8 and 12 gRNAs. (**a**) Sequences of the HBV X gene present in a chromosome of Gx11 cells. Blue bold line, X gene coding region. Three nucleotides differing between the two patients are shown as vertical boxes containing the diverse nucleotides in red. The blue numbers 2, 4, 5, 8, and 9 with bold and red vertical arrows show cut sites by Cas9. Their 20 nt recognition sequences are boxed in red. The black numbers 1, 3, 6, and 7 with thin and broken vertical arrows show the sites not cleaved by Cas9. Their 20 nt recognition sequences are boxed in black over and under the sequences. The PCR primers used for (**b**) are indicated at the top and bottom of this figure. (**b**) Multiple cleavages of the X gene using AdVs expressing 8 and 12 gRNAs. (Upper) Cleavage patterns using Axg8HBV-X. Gx11 cells were infected with Axg8HBV-X together with AxCBCas9 (AxCas9), and 3 days later, total cellular DNA was extracted and PCR was performed using the primer set shown in (**a**). The rates of disruption of the original 0.55 kb DNA using conventional PCR, which detects only deletions but not indels, are shown under the lanes. (Lower) Cleavage patterns using Axg12HBV-X-DR1. The representations are the same as above. Asterisks indicate the region consisting of close bands from 0.47 kb to 0.51 kb shown in (**c**). (**c**) Schematic explanation of the PCR fragments produced by 8 and 12 gRNAs expressed by AdVs. The PCR fragment of 0.55 kb without deletion is shown at the top, the positions of active cut 2, cut 4, cut 5, and cut 8 using Axg8HBV-X are shown in blue characters, and the position of cut 9, an extra cleavage site using Axg12HBV-X-DR1, is indicated in red character. The produced DNA fragments are shown below the top line together with their sizes. Among them, cut 2 and cut 8 were major features at high MOI, and the 0.44 kb fragment produced by them is shown as a bold line. Cut 1, cut 3, cut 6, and cut 7 shown in small green characters on the top line are uncleaved sites, with the exception of cut 1, which showed partial cleavage probably due to a strong off-target effect. The possible 0.54 kb fragment produced by double cleavages of cut 4 and cut 5, the second fragment of gray in the figure, was not detected. The reason for this may be that their cutting sites overlap ((a), fourth sequence row) and one cleavage abolished the 20 nt recognition sequence of the other. All other expected fragments were detected.

As a model of HCC containing diverse HBV DNA in a chromosome, we used the cell line Gx11 [30], which is derived from HepG2 cells containing the entire HBV X coding region integrated in a chromosome. Figure 2a shows the nucleotide sequences of the X gene. All four AdVs expressing multiplex gRNAs shown in Figure 1a are designed to target the C-terminal and central region of the HBV X gene shown in Figure 2a. The Axg8HBV-X shown as the second AdV in Figure 1a contains eight gRNA units that target the HBV-X central region at the eight possible cleavage sites (Figure 2a, sequence rows 4 and 5, vertical arrows from cut 1 to cut 8). The third AdV, Axg12HBV-X-DR1, in Figure 1a contains all twelve gRNA units and can additionally cleave at cut 9 (Figure 2a, sequence raw 7); the remaining three cleavage sites are not included in Gx11 cells. The sequences of all nine gRNA targets are shown in Supplementary Table S1a.

The recognition sequences of the nine gRNAs expressed by these AdVs and the target sequences of the HBV X gene in Gx11 cells are derived from different patients. There were three nucleotide differences, although the HBV genomes of the two patients belonged to common genotype C. They were A/T mismatch 2 nt upstream of cut 1, A/G between cut 3 and cut 4, and T/C between cut 6 and cut 7 (Figure 1b, fourth and fifth rows, vertical black boxes containing mismatch nucleotides). These three nucleotide differences were present in the 20 nt gRNA recognition sequences and may abolish the cleavages of cut 1, cut 3, cut 6, and cut 7 (black numbers 1, 3, 6, and 7 on the broken vertical arrows and overlapping open, thin horizontal boxes of 20 nt under or over the sequences, which contain the three mismatch nucleotides), while these three mismatches did not overlap with the gRNA recognition sequences of cut 2, cut 4, cut 5, cut 8, and cut 9 and may not affect their cleavages (blue numbers 2, 4, 5, 8, and 9 on the red vertical arrows and red horizontal boxes of 20 nt sequences filled in yellow). Therefore, the X gene may be cleaved only at four sites of cut 2, cut 4, cut 5, and cut 8 using Axg8HBV-X and at five sites including the additional site of cut 9 using Axg12HBV-X-DR1.

Gx11 cells were coinfected with Axg8HBV-X or Axg12HBV-X-DR1 at multiplicity of infection (MOI) of 0, 1, 3, and 10 together with AxCBCas9 [29] (AxCas9, hereafter), highly expressing Cas9, at the same MOI. Three days after infection, total cellular DNAs were extracted and authentic PCR was performed using a set of primers indicated in Figure 2a. The obtained results are shown in Figure 2b. Note that this assay detects only deletion between two or more cutting sites and does not detect small insertions/deletions (indels), although indel generation is the main mechanism of target disruption. When Axg8HBV-X was used, the intensity of the intact 0.55 kb band decreased depending on the MOI and the deletion efficiency was 92% at MOI 10 (Figure 2b, upper, lane 10). The main 0.44 kb DNA is the product of double cleavages of cut 2 and cut 8 and subsequent end-joining (Figure 2a, fourth and fifth rows). A significant amount of DNA was observed in the broad region between the 0.55 kb and 0.44 kb bands (shown as an asterisk). The region appeared to be a mixture of partial cleavage products that consisted of DNAs between cut 2 and cut 4, cut 2 and cut 5, cut 4 and cut 8, and cut 5 and cut 8 because their sizes exactly matched the expected sizes (illustrated in Figure 2c). Because the amount of DNA in the broad region is significant, these multiple partial digestions contributed to increase the disruption efficiency, showing the advantage of multiplex gRNA expression compared with the double cleavages of cut 2 and cut 8. Furthermore, although the 0.44 kb DNA fragment was abundant at MOI 10, the result does not necessarily mean that cut 2 and cut 8 were more effective than other cut sites because the 0.44 kb DNA is the final product produced from complete cleavage. However, the possibility that the cleavages of both ends are generally more efficient than the others cannot be ruled out. The band of approximately 0.43 kb was unexpected, but the DNA fragment was probably produced by the cleavage of cut 1 together with cut 8, suggesting strong off-target cleavage of cut 1.

Axg12HBV-X-DR1 additionally possesses the gRNA unit targeting cut 9 (Figure 2a, red “12 gRNAs” on the seventh row). Four additional bands of the predicted sizes derived from cut 9 in combination with cut 2, cut 4, cut 5, and cut 8 were observed (Figure 2c), and no other bands were detected. The results of partial cleavages using Axg8HBV-X and Axg12HBV-X-DR1 showed that all five gRNA expression units in tandem produced functional gRNAs. The disruption efficiency of the 0.55 kb band using Axg12HBV-X-DR1 was weaker than that of Axg8HBV-X. This result is expected because the AdVs that contained all twelve expression units were decreased (Figure 1c, “12 gRNAs”).

As noted above, these experiments using authentic PCR show only the efficiency of deletion produced by simultaneous cleavages and miss the disruption by small indels detectable by T7EI assay. This was confirmed using Axg4HBV-DR1, which cuts only a single site at cut 9. Although no band below 0.55 kb was detected using PCR deletion assay (Supplementary Figure S1, left), indel mutations at the target site were actually detectable as approximately 3.4% at MOI 10 by T7EI assay using the single gRNA (Supplementary Figure S1, right). Therefore, while the deletion efficiencies were 92% and 73% using Axg8HBV-X and Axg12HBV-X-DR1, respectively, those using eight and nine gRNAs would probably be even higher.

### 2.3. Southern Analyses Using the AdV Expressing Eight gRNAs Targeting HBV X Gene

**Figure 3:**
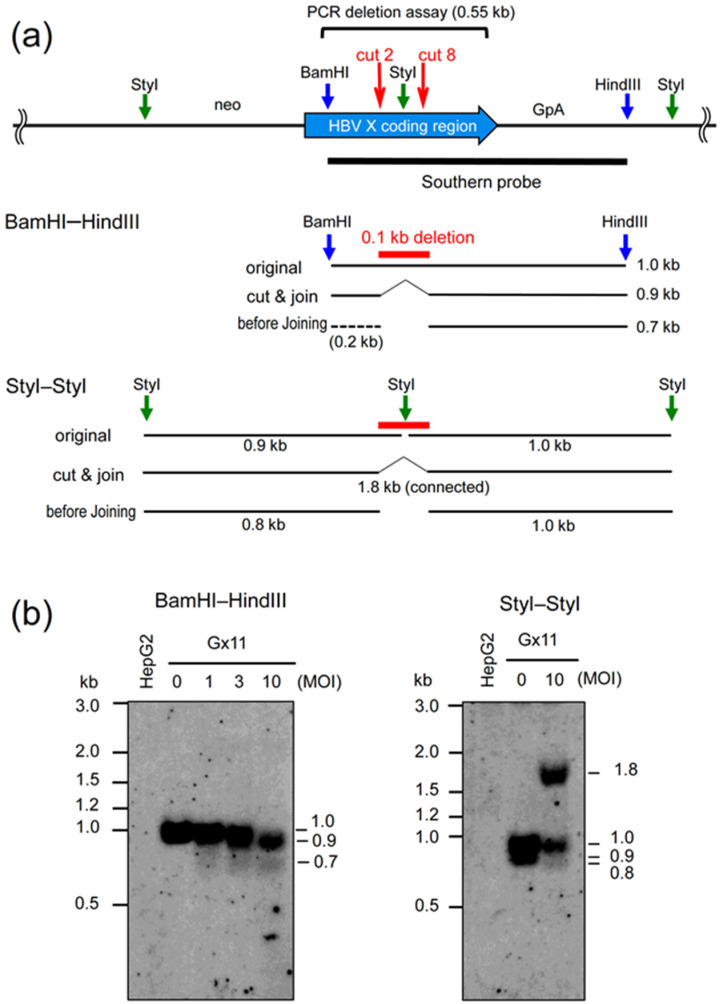
Southern analyses of HBV X gene integrated in Gx11 cell chromosome disrupted by the AdV expressing eight gRNA units. (**a**) The deleted region of the HBV X gene. All cleaved positions and the ends of the produced fragments are common among the upper, middle, and lower parts. (Upper) Cleavage sites of Cas9 and restriction enzymes used for Southern experiments at the HBV X coding region. The cleavage sites of cut 2 and cut 8 are shown as red vertical arrows on the HBV X coding region of the bold horizontal arrow. The 0.55 kb fragment amplified in the PCR deletion assay is shown above the arrow of the HBV X coding region; the range of the Southern probe (BamHI–HindIII fragment) is also shown below the HBV X arrow. neo, neo gene used for the selection of Gx11 cells; GpA, poly(A) sequences of the rabbit β-globin gene. These sequences are present because the integrated DNA fragments are derived from the pCAGGS plasmid. (Middle) The positions of the BamHI–HindIII fragment containing the deletion between cut 2 and cut 8. Because the same enzymes BamHI and HindIII are used for preparation of the Southern probe and for cleavages of total cell DNA, their terminal positions are the same. Expected sizes of the fragments in Southern analysis are shown on the right in kb. (Lower) The positions of the StyI–StyI fragment containing the deletion between cut 2 and cut 8. (**b**) The results of Southern analyses. Gx11 cells were infected with Axg8HBV-X at the indicated MOIs together with AxCas9 at the same MOI.

To confirm the deletion produced by Axg8HBV-X, we examined the disrupted structure of the HBV X gene integrated into a cell chromosome. Amplicon sequencing analysis is more sensitive and quantitative, but we chose Southern analysis to detect the deletion more directly without PCR amplification. Figure 3a (upper) shows the HBV X coding region (bold, blue arrow) and the surrounding areas. The major cleavage sites of cut 2 and cut 8 in the coding region are approximately 0.1 kb apart from each other (Figure 2a). Gx11 cells were infected with Axg8HBV-X at MOIs 1, 3, and 10, together with AxCas9 under the same condition as that in the PCR deletion assay shown in Figure 2b (upper). Then, the total cellular DNA was extracted and double-digested with BamHI and HindIII (blue vertical arrows in upper and middle panels). After the gel electrophoresis, Southern analysis was performed using the probe from BamHI to HindIII (black horizontal line under the arrow of the HBV X coding region).

The original 1.0 kb band shifted to 0.9 kb at MOI 10, which is 0.1 kb shorter. This indicated that the original BamHI–HindIII of 1.0 kb DNA was cleaved at cut 2 and cut 8 and the termini were end-joined, producing 0.9 kb DNA (Figure 3b, left panel, lane 10; explanations are shown in Figure 3a, middle panel, BamHI–HindIII, “original” and “cut & join”). This result also corresponded well with that obtained in the PCR deletion assay, where the amount of original 0.55 kb DNA decreased by 92% and the 0.44 kb DNA increased (Figure 2b, upper). Interestingly, a broad band of approximately 0.7 kb was also observed at MOIs of 1, 3, and 10 (Figure 3b, left panel, lanes 1, 3, and 10). This may represent the DNA fragments produced by Cas9 cleavages before end-joining (shown as a line of “before joining” at 0.7 kb). They cannot be detected by PCR, even if they are present, because PCR detects only the connected DNAs.

To confirm the deletion between cut 2 and cut 8 and also the presence of the possible termini before end-joining, the infected Gx11 DNA was also digested with StyI (Figure 3a, upper, green vertical arrows between cut 2 and cut 8 and near both terminals of the genome line). Because the StyI site is between cut 2 and cut 8, the 0.1 kb deletion by cut 2 and cut 8 removes it, and the end-joining must connect the original two StyI fragments of 0.9 kb and 1.0 kb, generating a large 1.8 kb fragment (Figure 3a, lower, “original” and “cut & join”). In fact, the expected 1.8 kb fragment was observed, and a broad band of 0.8 kb was also detected at MOI 10, confirming the presence of the “before-joining” DNA fragment by Southern analysis (Figure 3b, right panel).

## 3. Discussion

In this study, we showed that eight multiplex gRNA expression units were stably maintained in the AdV genome. Using this AdV, we showed that the HBV X DNA present in a cellular chromosome derived from a heterogeneous patient could be simultaneously cleaved at four or five expected sites, producing irreversible deletions. The results suggest that, although the HBV DNA sequences are highly diverse, a single AdV could be used as a possible anti-HCC drug for genome-editing therapy, when cleavage of the cell chromosome at the integration sites of HBV DNA is effective. The same AdV may also be applied for treating chronic or acute HBV hepatitis.

While we report AdVs containing eight gRNA units, this number is much greater than that using other viral vectors: four multiplex gRNAs have been reported using lentiviral [31], AAV [32], and adenoviral vectors [29]. AdVs containing eight gRNA units can be constructed using the standard method for the generation and amplification of AdVs. Therefore, the limiting step is not AdV construction and handling, but rather the construction of plasmids containing a >30 kb AdV genome with eight gRNA units with lack of deletion. We recently developed a method, Tetraplex-guide Tandem [29,33], and completely stable cosmids containing AdV genome bearing eight multiplex gRNA units were obtained in a single step utilizing lambda in vitro packaging. The AdVs that we used are commercially available Adex vectors [34,35], which possess the full Ad5 packaging domain [36], while other AdVs, for example, those of Bett et al. [37] and Mizuguchi et al. [38], do not. It is necessary to examine whether AdVs without lack of deletion of eight gRNA units can be obtained using other systems.

AdVs simultaneously expressing highly multiplex gRNAs may be useful for effective and reliable knockout of multiple target genes. The AdVs expressing eight gRNAs can simultaneously knockout up to eight genes. It has been reported that two adjacent gRNAs targeting one locus significantly facilitate gene disruption using native Cas9 [39]. Furthermore, it is often difficult to study the function of a gene owing to the presence of a family of genes possessing similar function.

Simultaneous expression of eight multiplex gRNAs by AdVs has more merits than that of four gRNAs, which we reported previously [29]. The method of Tetraplex-guide Tandem can be used to construct an array of four or eight gRNA expression units in one step, and the construction of other numbers of gRNA units requires some modification. Although AdVs containing four or eight units can be constructed similarly in one step, most of the AdVs containing multiplex gRNA units produced in our lab are eight-unit AdVs using double-nicking strategy. Off-target effects are an important concern when using native Cas9. When more multiplex gRNAs are expressed, the off-target effects may increase in proportion. For example, in the present study, strong off-target cleavage was observed using the gRNA of cut 1 (Figure 2b, upper and lower, 0.43 kb band). Multiplex gRNA expression enables us to use a double-nicking strategy with Cas9 nickase [29,40,41], which decreases off-target effects by up to 1500-fold. This strategy offers safer genome-editing therapy and is preferable for all experiments using the CRISPR/Cas9 system. The cleavage efficiency of double-nicking is no less than that of single cleavage using native Cas9, and is sometimes higher [29,42]. However, in this strategy, two gRNAs are needed for one cleavage, where two nicks are introduced at sense and antisense strands preferably within 30 nt. Notably, a single cleavage using double-nicking does not produce small indels but irreversible deletion of several to several dozen base pairs in length [40,43]; therefore, complete disruption can be achieved.

A problem of the double-nicking strategy is that both of the two gRNAs for one cleavage must be active, so the possibility of low cleavage efficiency increases. It is not practical to assay the activity of all of the candidate gRNAs before the experiment, and there may be a position effect that influences the gRNA activity. Therefore, it is safe to use two double-nicking cleavages for one target. Moreover, the knockout of two sites for one gene or simultaneous disruption of two genes is effective to inactivate the function of a cascade, if available. For these reasons, eight-unit AdVs are preferable. Of course, four genes could be disrupted simultaneously using double-nicking. When the target genes are heterogeneous, as in the present study, AdVs that express eight multiplex gRNAs are obviously advantageous. The use of this strategy with the AAV vector is difficult because the essential PAM sequence of saCas9, NNGRRT, is less frequent than that of spCas9, NGG [44].

Simultaneous expression of eight gRNAs could be sufficient to introduce irreversible deletions at three or four sites in the HBV genomes of major genotypes using the double-nicking strategy. In HCC caused by HBV, multiple integrated viral DNAs are often present in the single or multiple chromosomes in the same cell. Very large deletion between two viral DNAs in one chromosome or damages in two or more chromosomes produced might affect viability of these HCC cells using the multiplex gRNA approach, if two targets, at least, are present in one chromosome, and even if viral DNA sequences are heterogeneous. AdVs expressing highly multiplex gRNAs may be used for other viruses possessing a DNA genome, for example, human immunodeficiency virus (HIV). Although genome-editing therapy of HIV has been extensively studied, their viral genomes are also very diverse [21,45]. Lebbink reported that combinatorial approaches using two potent gRNAs effectively prevent viral escape, but inefficient cleavage of one of the two gRNAs was not effective, probably because irreversible deletion of the HIV genome was not achieved [19]. We consider that the approach using eight multiplex gRNA expression units could be useful. Although commonly used AdV type 5 shows low infectivity of T cells, AdVs of other serotypes could be employed [46]. Moreover, it should be taken into account in genome-editing therapies using saCas9 and spCas9 that most of possible patients may possess preexisting adaptive immunity to these enzymes [26,27,28].

It is unexpected that the ends of the cleaved cell chromosome produced by native Cas9 before end-joining were detected in Southern experiments. This probably occurred because the Cas9 cleavage efficiency in our system is sufficiently high to detect these molecules. PCR detects only the end-joined DNA, so the presence of the ends before joining could have been missed. This suggests that two ends produced by one cleavage of Cas9 may not immediately be joined by the cellular machinery. We imagine that the detected ends of Cas9-cleaved DNA may not be free end products present in the nucleus, but be short-lived, transient products included in a complex consisting of Cas9, gRNA, and target DNA. The relationship of these ends with chromosomal rearrangement produced by Cas9 is unknown.

In conclusion, we showed that AdVs expressing eight multiplex gRNAs were stable. Although it is necessary to examine whether these AdVs can be stably amplified to a scale sufficient for therapeutic purposes, they could be applied for genome-editing therapy of HBV HCC and chronic hepatitis. They are also probably useful for basic studies, in which simultaneous knockout of multiple genes is desirable. Cassette plasmids of the improved version of the method of Tetraplex-guide Tandem will become available from Addgene, and the AdV expressing Cas9 under the control of CB promoter, AxCBCas9, will be available from RIKEN BioResorce Bank (https://dna.brc.riken.jp/en/rvd/adenoen, Clone Search, accessed on 28 September 2021).

## 4. Materials and Methods

### 4.1. Cell Culture

The human 293 cell line and HepG2 cell lines are derived from human embryonic kidney and human hepatocellular carcinoma, respectively. Gx11 is a cell line derived from HepG2, which possesses the HBV X gene in an inactive state [30]. The Gx11 cell line was obtained from Dr. Kazuhiko Koike (University of Tokyo, Tokyo, Japan). The 293 cells were cultured in Dulbecco’s Modified Eagles Medium (DMEM) (Kohjin Bio, Co., Ltd., Saitama, Japan) supplemented with 10% fetal calf serum (FCS). The 293 cells constitutively express adenoviral E1 genes and support the replication of E1-substituted AdVs. The HepG2 and Gx11 cells were kept in high-glucose DMEM (Kohjin Bio) supplemented with 10% FCS.

### 4.2. Construction of Cosmids Containing AdV Genome Bearing Multiplex gRNA Units

Multiplex gRNA expression units were constructed in accordance with the Tetraplex-guide Tandem method [29]. For the construction of eight gRNA units targeting the central HBV-X region, two fragments of head block and mid block, each containing four gRNA units, were doubly cleaved with AlwNI and PvuII and cloned into the SwaI site of pAxc4wit2 [47] as described previously [29] with slight modification. pAxc4wit2 is a derivative of pAxcwit2, which is commercially available from Takara Bio (Shiga, Japan) or RIKEN Bioresource Center (RIKEN BRC: https://dna.brc.riken.jp/en/key_search?q=pAxcwit2, accessed on 28 September 2021). The former contains an SwaI site in the E4 region, and the latter contains it in the E1 region. The multiplex gRNA units can be cloned at the SwaI site in either cosmid. The SgrDI sequence, 5′-CGTCGACG-3′, was added into the *ampl Head-A F primer, so that the 5′ terminal of eight gRNA units possessed the SgrDI site, which is not present in the AdV backbone. The cosmids containing 12 and 16 gRNA units were constructed by inserting a fragment containing four gRNA units of DR1 into the unique SgrDI site.

### 4.3. Production of AdVs

AdVs were produced as previously described [29]. Briefly, 293 cells were transfected with the BstBI-linearized AdV genome in pAxc4wit2, using Lipofectamine LTX with Plus Reagent (Thermo Fisher Scientific, Inc., Waltham, MA, USA), and the next day, the cells were transferred to a 96-well plate. The first viral stocks were obtained within 2 weeks (approximately 150 µL). Cells in the 24-well plates were infected with half of the first stock to obtain the second stock, and one-tenth of the second stock was used for the third stock. The amount of stock solution used for further stock amplification was 2- or 3-fold more than usual, while overinfection caused toxicity and was carefully avoided. Total cellular DNA of the second, third, and fourth stocks was isolated and subjected to BspEI digestion, as described previously [29]. The vector DNA fragment could be seen by agarose gel electrophoresis without separation of the cellular DNA because the copy numbers of the AdV genome were very high and the BspEI recognition sequence contained CG dinucleotide, which is uncommon in mammalian DNA. AdVs were titrated using a method described previously [47,48]. Briefly, the copy numbers of the viral genome that was successfully transduced into the infected HepG2 cells were measured by real-time PCR. The TCID50r in this work is the same as the relative viral titer (rVT) in these references based on the TCID50 titer of control virus AxEFGFP. The rTV titers are normally equivalent to TCID50 and PFU, but more accurately reflect the copy numbers introduced into the target cells because they are not influenced by the gene product that may be toxic to 293 cells.

### 4.4. Conventional PCR

Gx11 cells in 24-well cell culture dishes were infected at MOI 1, 3, and 10 and incubated for 3 days postinfection in DMEM supplemented with 5% FCS. The same amount of AdV expressing multiplex gRNAs and native Cas9, AxCBCas9 (AxCas9) [29], was used for infection. Total cellular DNAs were prepared and amplified by PCR with Tks Gflex DNA polymerase (Takara Bio) using the primer set HBV-X F (5′-AACTGGATCCTGCGCGGGACGTCCTTTGTC-3′) and HBV-X R (5′-GAAAAAGATCTCAGTGGTATTTGTGAGCCAGG-3′). The PCR cycling conditions were as follows: 94 °C for 1 min, followed by 30 cycles at 98 °C for 10 s, 65 °C for 15 s, and 68 °C for 30 s. Images of DNA agarose gels after electrophoresis were recorded using Printgraph AE-6932GXES (ATTO, Tokyo, Japan) and were analyzed using the software Image J 1.52a version [49]. The PCR products of the original 0.55 kb DNA were quantified, and the deletion efficiency was calculated as ratios against MOI 0.

### 4.5. T7 Endonuclease Assay

The T7EI assay was performed as described previously [29]. Briefly, an amplified PCR product was reannealed to form a heteroduplex using the following program: denaturation at 95 °C for 5 min, reannealing from 95 °C to 85 °C at −2 °C/s, holding at 85 °C for 1 min, cooling from 85 °C to 25 °C at −0.1 °C/s, and holding at 25 °C for 1 min, followed by cooling to 4 °C. The sample was then exposed to T7 endonuclease I (New England BioLabs Japan Inc., Tokyo, Japan) at 37 °C for 15 min and analyzed on agarose gels. These gels were imaged and quantified by ImageJ.

### 4.6. Cellular DNA Isolation

For the preparation of total cellular DNA, cells were suspended and incubated in a lysis buffer containing 10 mM Tris-HCl (pH 8.0), 150 mM NaCl, 10 mM EDTA, 100 µg/mL proteinase K, 80 µg/mL RNase A, and 0.1% SDS at 50 °C for 2 h. The mixture was extracted twice with phenol/chloroform and twice with chloroform, and precipitated with two volumes of ethanol at −20 °C for 1 h and then washed once with 70% ethanol. The pellet was dissolved with TE buffer.

### 4.7. Southern Blot Assay

Total cellular DNAs (10 µg) were digested by BamHI/HindIII or StyI at 37 °C for 4 h and were subjected to agarose gel electrophoresis. The agarose gel was treated with 0.5 M NaOH solution, and the DNA was then transferred to the nylon membrane Hybond-N (GE Healthcare Life Sciences, Chalfont Saint Giles, UK) using the capillary-transfer method. The probe is derived from the full HBV genome labeled with digoxigenin-UTP using a DIG DNA Labeling and Detection Kit (Roche Diagnostics, Basel, Switzerland), and specific DNA was detected using the chemiluminescence of CDP-Star, in accordance with the manufacturer’s protocol. The bands were visualized using LAS-4000 (Fujifilm, Tokyo, Japan).

## Data Availability

The data that support the findings of this study are available from the corresponding author upon reasonable request.

## Supplementary Materials

The following are available online at https://www.mdpi.com/article/10.3390/ijms221910570/s1.
