# Supplementary material for: Adenovirus Vectors Expressing Eight Multiplex Guide RNAs of CRISPR/Cas9 Efficiently Disrupted Diverse Hepatitis B Virus Gene Derived from Heterogeneous Patient
Source: Int J Mol Sci. 2021 Sep 29;22(19):10570. doi: 10.3390/ijms221910570 (PMC8508944; doi:10.3390/ijms221910570)
Supplement: Supplementary file 1 [file ijms-22-10570-s001.zip › ijms-1385879-supplementary.pdf]

Supplementary Table S1

Sequences of target gRNAs in HBV DR1 region and X gene

gRNA	oligo name	sequence
cut-1	HBc 69b1	GAAGCGAAGTGCACACGGTC
cut-2	HBc 70b2	GAGGTGAAGCGAAGTGCACA
cut-3	HBc 71b3	GGTTTCCATGCGACGTGCAG
cut-4	HBc 72b4	GACCTGGTGGGCGTTCACGG
cut-5	HBc 73t1	GTCTTATATAAGAGGACTCT
cut-6	HBc 74b2	GACCTTGGGCAAGACCTGGT
cut-7	HBc 75b3	GTCCTCTTATATAAGACCTT
cut-8	HBc 76t4	GATGTCAACGACCGACCTTG
cut-9	HBc 80b1	GCAGAGGTGAAAAAGTTGCA

In addition to the cut numbers in Figure 2a, the original name of oligos and their sequences are shown. The sequences are the same as those in Supplementary Figure S3 in Nakanishi T *et al.*, J Gene Med, 21: e3115, 2019, on the sequences of the HBV X gene.

Supplementary Figure S1

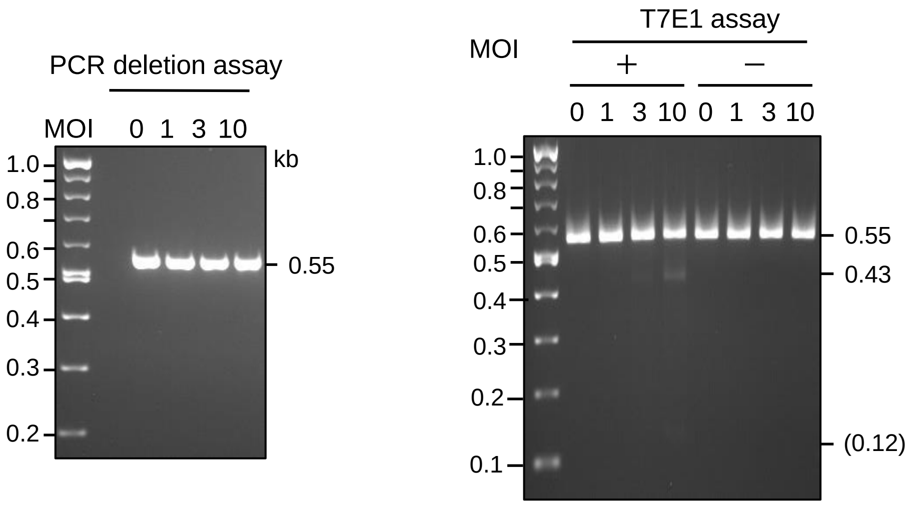

Confirmation of the difference between PCR deletion assay and T7E1 assay. It was expected that a single cleavage would not be detected by the PCR deletion assay, but would be detected by the T7E1 assay. To confirm this, Gx11 cells were coinfected with Axg4HBV-DR1 and AxCBCas9 at the indicated MOIs. This AdV expresses the one gRNA (cut 9) that targets the HBV X gene. Three days later, total cellular DNA was prepared and both assays were performed. No band was detected using the PCR deletion assay (left panel) and the expected 0.43-kb band was observed at MOI 10 using the T7E1 assay (right panel).
